# A case control study of cardiovascular health in chemical war disabled Iranian victims

**DOI:** 10.4103/0972-5229.74168

**Published:** 2010

**Authors:** Atoosheh Rohani, Vahid Akbari, Fatemeh Tabesh Moghadam

**Affiliations:** **From:** Yasuj university of medical science, Yasooj, Iran; 1 Internal medicine ward, Sajjad Hospital, Yasooj, Iran

**Keywords:** Cardiovascular abnormalities, chemical warfare, mustard gas

## Abstract

**Background:**

Sulfur mustard (SM) is an alkylating chemical warfare agent that was widely used during Iran–Iraq war between 1983 and 1988. SM exposure leads to various late complications. The aim of this study was to determine the late cardiovascular effects of SM in war-disabled Iranian victims.

**Materials and Methods:**

This was a retrospective cohort case control study on 50 patients with symptoms of SM exposure and 50 cases who had been in Iran–Iraq war, without chemical injury. We performed exercise stress test and echocardiography for all of patients.

**Results:**

The study group comprised 100 males of mean age 45.6 ± 6.2 years. In chemical war injury group, two patients (4%) had positive exercise stress test. On coronary angiography, they were found to have coronary artery disease. One patient had severe mitral regurgitation and normal coronary angiography; he was referred for mitral valve replacement. Left ventricular (LV) diastolic abnormality was detected in 23% of these subjects. In another group, 5% had LV diastolic abnormality (*P* = 0.02) and all of them had normal stress test.

**Conclusions:**

Cardiovascular abnormalities are another late complication in chemical war disabled Iranian victims. Diastolic dysfunction was the most common abnormality in both groups of patients.

## Introduction

Sulfur mustard (SM) is an alkylating chemical warfare agent that was widely used during Iran–Iraq war between 1983 and 1988.[1] Today, the actual number of victims is estimated to be more than 100,000, since the long-term effects still cause casualties.[2] The official estimate does not include the civilian population residing in bordering towns or the children and relatives of veterans, many of whom have developed blood, lung and skin complications, according to the Organization for Veterans of Iran. Today, more than 20 years post war, enormous number of Iranians, both military veterans and civilians, are afflicted with medical problems arising from exposure to Iraqi chemical weapon. Mustard (blistering) agents were the most widely used chemical agents in the Iran–Iraq war.[2]

These agents are known to cause both acute toxic effects and long-term medical complications which are widely investigated, although the best known and the most severe annoying complications of exposure to these agents are those related to respiratory, ocular and skin.[3] To our knowledge, so far, there have been few earlier reports concerning the long-term cardiac effects of these agents.[4–7] However, their cardiovascular effects are still unclear. The aim of this study was to determine the late cardiovascular effects of SM in war-disabled Iranian victims.

## Materials and Methods

We performed a study on a population of patients who were consecutively referred to our cardiology clinic by their physicians for evaluation of the cardiovascular system between 15th May 2007 and 15th October 2009. The investigation conforms to the principles outlined in the Declaration of Helsinki. All patients were symptomatic. We found 50 consecutive cases who claimed to have had a history of intoxication by chemical warfare agents. After an attending interview of these patients, the outline of the project was explained and their verbal consent was obtained. Thereafter, the past medical records of these patients and also other official documents concerning their intoxication during the war were evaluated, and only those patients who met the eligibility criteria [Table 1] were subsequently enrolled in the final analysis. Fifty participants had evidence of mustard intoxication and their echocardiography and exercise stress test findings were compared with a group of 50 non-intoxicated control subjects from a similar population (they had been involved in the war but were not affected by chemical agents). We could not measure the exposure dose and severity because the gas spreads through an indefinite area. None of these cases and also controls had other medical problems such as diabetes, hypertension, smoking or positive family history of any cardiac diseases.

**Table 1:** Eligibility criteria for entering into the study as a warfare patient

Inclusion criteria
Participants with documented evidence of mustard intoxication during the war No known cardiac disorders or positive family history of any cardiac diseases Presence of other confirmed complications of intoxication Moderate to severe respiratory dysfunction Cutaneous complications Cutaneous complications
**Exclusion criteria**
Patients intoxicated with nerve agents Those who have known any cardiovascular diseases

Patients with prior history of mediastinal or spinal radiation, chemotherapy (that are known to be associated with chronic or late cardiotoxic effects) and history of rheumatic or congenital heart disease were excluded. No patient had known hypothyroidism at the time of the echocardiographic evaluation.

At that time, none of the participants were taking any cardiac medication. Only those mustard intoxicated patients were enrolled in the study, who had currently other confirmed complications of intoxication (i.e. moderate to severe respiratory dysfunction[4,8–12] and cutaneous[13] or ocular complications).[14] Exercise stress test and echocardiography were performed on them by two cardiologists, whoere blinded to other clinical data, and the final diagnoses were reached by consensus. The echocardiographic study consisted of a two-dimensional echocardiogram and Doppler evaluation with stress–velocity analysis, which were obtained through parasternal long- and short-axes, apical four-chamber views. During echocardiography, the pericardium, valvular anatomy and function, left- and right-sided chamber size, and cardiac function were also assessed. Impaired relaxation of left ventricle (LV) was defined as an increase of the late atrial filling phase so that (E wave)/(A wave) ratios on the mitral Doppler pattern declined. Furthermore, physical examination, electrocardiogram, and exercise stress test were performed. If there was any contraindication to stress test (unstable angina, uncontrolled hypertension) or patient was not able to undergo the test, (orthopedic problem), the patient was excluded from the study. Patients underwent an exercise treadmill test (ETT) using Bruce protocol.[15] A semi-quantitative treadmill score was derived, with ETT being considered as 1) positive: horizontal or downsloping ST segment depression of 1–2 mm measured 0.08 second after the J point, occurring for a workload ≤5 METs, with or without chest pain; 2) strongly positive: ST segment depression >2 mm at any workload, post-exercise ST depression for a duration >6 minutes; 3) negative: when ST segment remained isoelectric and heart rate ≥85% of the maximum age-predicted heart rate was achieved; and 4) nondiagnostic in all other cases. Patients who did not achieve 85% of age-related work load were defined as incomplete EET. Stress test was continued till the patients achieved 85% maximum heart rate, except in patients with high risk criteria.[15]

### 

#### Statistical analysis

Quantitative data were expressed as mean ± SD. Analysis of variance (ANOVA) test was used to calculate the significance of the differences between the quantitative and the qualitative variables. We performed the Student *t*-test to assess the differences between quantitative variables and the chi-square test for contingency tables of qualitative variables. A *P* value of less than 0.05 was considered to indicate a statistically significant difference for all compared variables. SPSS for Windows software package (Release 15, SPSS) was used for statistical analysis.

## Results

The study group comprised 100 males of age 45.6 ± 6.2 years. Their mean weight was 67.2 ± 8.3 kg. SM poisoning was confirmed 21.5 ± 1.6 (21–24) years after the initial exposure.

The most common complication was found in the lungs (100%), skin (82.84%), and eyes (77.61%). Typical chest pain was more frequent in the exposed group (5% vs. 0%, *P* = 0.450); atypical chest pain was equal in both the groups (22% vs. 20%, *P* = 0.2). In addition, exertional dyspnea was found in 50% of the exposed group versus 30% in the control group (*P* = 0.162). Chest pain and dyspnea were more frequent in the exposed group and cardiac signs were the same in both the groups. In chemical war injury group, two patients (4%) had highly positive exercise stress test, and in coronary angiography, one of them had 2-vessel disease and another had 3-vessel disease, whereas ejection fraction was normal in both of them. They both underwent successful angioplasty. One patient developed anterior wall myocardial infarction before exercise stress test, in coronary angiography, he had 3-vessel disease, and unfortunately died after coronary artery bypass graft (CABG) (45 years old). LV diastolic abnormality (relaxation impairment) was detected in 23% of these subjects. The average LV diastolic dimension was 5.4 ± 0.92 cm in chemical patients and it was 4.3 ± 0.73 in the control group (*P* = 0.001). The average LV systolic dimensions were 4.7 ± 0. 43 in warfare victims and 3.6 ± 0.45 in the control group (*P* = 0.02). The average ejection fraction (according to Simpson formula) was 45 ± 2.6% in warfare victims and 53 ± 3.5% is EF in control group (*P* = 0. 01). The average right ventricular (RV) diastolic dimension was 3.4 ± 0.6 in the patients and it was 2.3 ± 0.6 in the control group (*P* = 0.02). The E:A ratio was 0.85 ± 0.2 in chemical warfare patients and 1.02 ± 0.35 in the control group. The E wave deceleration time was 0.11 ± 0.02 seconds in chemical veterans versus 0.17 ± 0.04 seconds in the control group.

There were no considerable conductive abnormalities. One of the patient in chemical war injury group had severe mitral regurgitation and normal coronary angiography and he was referred for mitral valve replacement. In another group, there were no considerable valvular or conductive abnormalities. About 5% of them had LV diastolic abnormality (relaxation impairment). (*P* = 0.02); all of them had normal exercise stress test.

## Discussion

According to our study, cardiovascular disability is a late complication in chemical war disabled Iranian victims. It seems that some of these findings are similar to the echocardiographic pattern of dilated cardiomyopathy, however, in a minor form or severity. This preliminary hypothesis about the potential of mustard agents in developing some degree of dilated cardiomyopathy is based on significantly higher LV diastolic and systolic dimensions as well as lower ejection fraction. Another interesting finding in these victims was higher RV size dimensions. It may be due to the well-known phenomenon of cor pulmonale secondary to their lung problem (chronic bronchitis, bronchiectasis and lung fibrosis), but it emphasizes again on the importance of evaluation of the cardiac performance in these patients.

We must emphasize on the management of RV failure because it can play a significant role in the patient’s dyspnea.

In a study by Gholamrezanezhad *et al*.,[6] the results of scintigraphic myocardial perfusion scans in 22 mustard intoxicated patients and in 14 controls revealed a pattern of myocardial perfusion that was significantly different from controls. According to these data, further evaluation to exclude cardiomyopathic effects of mustard agents is extremely warranted.

Diastolic dysfunction is difficult to define clinically and in our study diastolic abnormality was the most common abnormality in both the groups. It may be due to ischemia (epicardial or microvascular) and cardiomyopathic effects of mustard agents because all patients with systolic dysfunction have concomitant diastolic dysfunction; therefore, a patient cannot have pure systolic heart failure. Transmission of higher end-diastolic pressure to the pulmonary circulation may cause pulmonary congestion, which leads to dyspnea and subsequent right-sided heart failure. It may be another explanation for RV failure in these patients. The incidence of diastolic heart failure increases with age; aging may be the cause of diastolic abnormality in the control group. Diastolic dysfunction may be present for several years before it is clinically evident. So, early diagnosis and treatment are important in preventing irreversible structural alterations and systolic dysfunction.

Premature coronary artery disease was another late complication of these patients. Although our group of patients consisted of those who had an extremely low risk of coronary artery disease, the final finding raises the possibility of increased prevalence of ischemic heart disease in these patients.

According to the study results, the stress test and coronary angiography were compatible with the ischemic component in three patients. Meanwhile, we have limited knowledge about the cardiovascular effects of sulfur mustard so, the pathogenesis of coronary artery disease in intoxicated patients might be multifactorial and it needs further investigation.

Exposure to SM causes pulmonary complications resulting in disability in affected patients.[16–19] Further research is necessary to measure the health-related quality of life in victims with different types of disabilities in order to support and enhance the quality of life amongst this population.

Research into the health consequences of SM must include follow-up in this group of patients due to the well-documented latent neuropathic,[16] pulmonary, cardiac, and carcinogenic and hematologic[18] effects. We recommend yearly screening, educating patients on the long-term effects of SM exposure and the use of prevention strategies such as immunization. It should be emphasized that if any cardiovascular complication (such as cardiomyopathy, coronary artery disease, or any other possible pathology) is present in these patients, it warrants careful attention and screening of these victims. The serum brain natriuretic peptide (BNP) test can accurately differentiate heart failure from noncardiac conditions in a patient with dyspnea; so, we recommend this test for these patients. As a consequence of the underlying severe pulmonary disease, in these patients, dyspnea and chest discomfort are commonly present and, therefore, the diagnosis or excluding the presence of underlying cardiovascular diseases could be considered. Although it is clear that pulmonary complications are the most important causes of morbidity in the mustard intoxicated victims,[18] according to our study findings, it should be kept in mind that possible cardiovascular complications can also play a relatively considerable role in these symptoms and disabilities. In addition, an autopsy should be performed in all cases where death is the outcome.[18,19] We should emphasize that one major limitation of our study was the limited number of patients analyzed. Therefore, the study findings must be addressed in a larger series of patients. Finally, it may be useful to instruct military experts to equip and train local chemical and biological response teams in order to establish strategies for inhibiting the production and use of chemical weapons. Ideally, we would like to see any remaining chemical weapons, existing around the world, safely destroyed.
